# Progranulin, a Glycoprotein Deficient in Frontotemporal Dementia, Is a Novel Substrate of Several Protein Disulfide Isomerase Family Proteins

**DOI:** 10.1371/journal.pone.0026454

**Published:** 2011-10-18

**Authors:** Sandra Almeida, Lijuan Zhou, Fen-Biao Gao

**Affiliations:** 1 Department of Neurology, University of Massachusetts Medical School, Worcester, Massachusetts, United States of America; 2 Gladstone Institute of Neurological Disease, San Francisco, California, United States of America; Thomas Jefferson University, United States of America

## Abstract

The reduced production or activity of the cysteine-rich glycoprotein progranulin is responsible for about 20% of cases of familial frontotemporal dementia. However, little is known about the molecular mechanisms that govern the level and secretion of progranulin. Here we show that progranulin is expressed in mouse cortical neurons and more prominently in mouse microglia in culture and is abundant in the endoplasmic reticulum (ER) and Golgi. Using chemical crosslinking, immunoprecipitation, and mass spectrometry, we found that progranulin is bound to a network of ER Ca^2+^-binding chaperones including BiP, calreticulin, GRP94, and four members of the protein disulfide isomerase (PDI) family. Loss of ERp57 inhibits progranulin secretion. Thus, progranulin is a novel substrate of several PDI family proteins and modulation of the ER chaperone network may be a therapeutic target for controlling progranulin secretion.

## Introduction

Frontotemporal lobar degeneration (FTLD), the most common cause of dementia before the age of 60 years [1], causes behavioral and cognitive abnormalities, and up to 40% of patients have a family history of dementia [1], [2]. Mutations in several genes have been implicated in the pathogenesis of familial FTLD, including progranulin (PGRN), a secreted protein whose functions in the nervous system are poorly understood [3], [4]. PRGN mutations are a major cause of the disease, and genetic evidence suggests that haploinsufficiency is involved, since disease-associated mutations in PGRN often lead to reduced PGRN production or activity [3]–[8]. It is not known how such reductions lead to neurodegeneration. However, molecular interventions that increase PGRN production or secretion from the remaining wildtype allele are a promising therapeutic strategy for FTLD caused by PGRN deficiency.

It is likely that the levels of intracellular and secreted PGRN are regulated through multiple mechanisms. PGRN transcription can be enhanced by several small molecules, including suberoylanilide hydroxamic acid, an FDA-approved histone deacetylase inhibitor [9]. PGRN production and secretion can also be modulated through post-transcriptional mechanisms. For instance, loss of microRNA-29b activity increases the levels of intracellular and secreted PGRN in 3T3 cells [10]. MicroRNA-107 also contributes to the regulation of PGRN expression [11]. Interestingly, a common genetic variant, rs5848, in the 3′ untranslated region of human PGRN (hPGRN) mRNA increases the binding and inhibitory activity of microRNA-659 [12]. Moreover, inhibitors of vacuolar ATPase and some clinically used alkalizing drugs increase PGRN production and secretion through a translational mechanism [13]. Extracellular levels of PGRN are also influenced by its rate of uptake through sortilin-mediated endocytosis [14], [15].

It is not known how PGRN, a secreted and highly glycosylated protein, is regulated in the endoplasmic reticulum (ER)-Golgi secretory pathway. hPGRN contains 7.5 granulin domains, each of which contains 12 cysteines that form six intramolecular disulfide bonds [16]. In mouse brain, mouse PGRN (mPGRN) expression increased more than 60-fold in activated microglia [17], posing a significant challenge for proper folding and efficient secretion. In this study, through biochemical approaches, we found that PGRN is a novel substrate of four protein disulfide isomerase (PDI) family proteins, raising the possibility that modulation of the ER chaperone network may be a therapeutic target for controlling progranulin secretion.

## Results

### Identification of PGRN-Interacting Proteins

**Figure 1:**
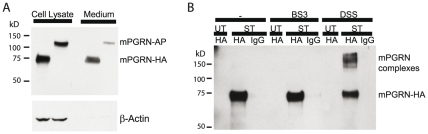
Biochemical identification of PGRN-interacting proteins. (**A**) mPGRN-AP or mPGRN-HA fusion protein constructs were stably transfected into HEK293 cells. Cell lysates or culture medium was analyzed on Western blot with anti-mPGRN antibody. This antibody specifically recognizes mPGRN but not the endogenous hPGRN in HEK293 cells. β-actin was used as the loading control. (**B**) Stably transfected HEK293 cells expressing mPGRN-HA were treated with cross linkers, followed by immunoprecipitation (IP) with HA antibody or control IgG. Immunoisolates were analyzed on Western blot with anti-mPGRN antibody. UT, untransfected HEK293 cells; ST, stably transfected cells.

To identify regulators of PGRN secretion and PGNR-interacting proteins, we constructed fusion proteins whose expression is controlled by the CMV promoter, including mPGRN-tagged with alkaline phosphatase (AP) or HA. We transfected these constructs into HEK293 cells (Invitrogen) and examined the levels of intracellular or secreted mPGRN by Western blotting with an mPGRN-specific antibody (R&D Systems) that does not recognize endogenous hPGRN in HEK293 cells. mPGRN-HA but not mPGRN-AP was effectively secreted into the culture medium even though both constructs contained the mPGRN signal peptide (Fig. 1A).

To facilitate the identification of mPGRN-interacting proteins, we established stably transfected HEK293 cells expressing mPGRN-HA. This system enabled us to examine the molecules that specifically regulate mPGRN secretion, since mPGRN-HA is controlled by the constitutively active CMV promoter, and endogenous hPGRN expression is sensitive to many extracellular stimuli including various transfection agents and is regulated both transcriptionally and posttranscriptionally (unpublished observation). Assuming that some proteins exhibit stable physical interactions with PGRN, we treated these cells with the membrane-impermeable chemical crosslinker bis(sulfosuccinimidyl) suberate (BS) and the membrane-permeable crosslinker DSS. With DSS but not BS treatment, a significant fraction of mPGRN showed a slower mobility as detected by Western blot (Fig. 1B). This experiment did not identify membrane surface proteins that might be bound with extracellular mPGRN but revealed a direct interaction between mPGRN and some proteins that are likely to be intracellular.

**Figure 2:**
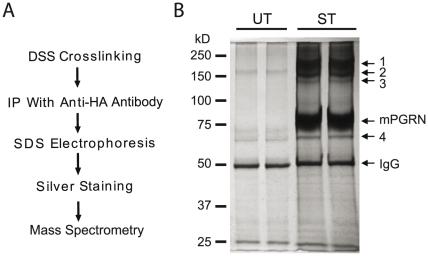
Biochemical identification of PGRN-interacting proteins. (**A**) Description of the experiment in *C*. mPGRN-HA stably transfected HEK293 cells were treated with the chemical crosslinker DSS. Immunoprecipitation was performed with an anti-HA antibody. The immunoisolates were analyzed by SDS-PAGE, which was then silver stained. Specific bands were cut out and analyzed by mass spectrometry. (**B**) Image of a gel after silver staining. The identities of bands 1–4 are listed in Table 1.

To identify these mPGRN-interacting proteins, we used HA antibody to immunoprecipitate mPGRN-containing protein complexes after DSS crosslinking and analyzed the proteins by SDS-PAGE and silver staining (Fig. 2A). It seems that some proteins were complexed with or crosslinked with mPGRN (Fig. 2B). The molecular sizes of bands 1–3 were very similar to that detected by Western blotting with mPGRN-specific antibody (Fig. 1B) and are therefore likely to be high-molecular-weight complexes containing proteins covalently crosslinked with mPGRN. Indeed, mass spectrometry analysis revealed multiple peptides corresponding to several major Ca^2+^-binding chaperone molecules residing in the ER, including BiP, calreticulin, GRP94, PDI, and the PDI-related proteins ERp57, ERp72, and ERp5 (Table 1). Band 4 was the molecular chaperone HSP70-1, which was also present in the crosslinked complex in band 3 (Table 1), indicating a direct interaction between mPGRN and HSP70-1.

**Table 1:** Identification of proteins that are crossed-linked with progranulin.

Band	Size	Identified Proteins
1	200 kD	BiP, Calreticulin, GRP94, PDI, ERp57, ERp72, ERp5
2	150 kD	BiP, Calreticulin, PDI, ERp57, ERp5
3	135 kD	BiP, Calreticulin, GRP94, HSP70-1, PDI, ERp57, ERp5
4	70 kD	HSP70-1

### PGRN Co-localizes with Several Markers in the Secretory Pathway

**Figure 3:**
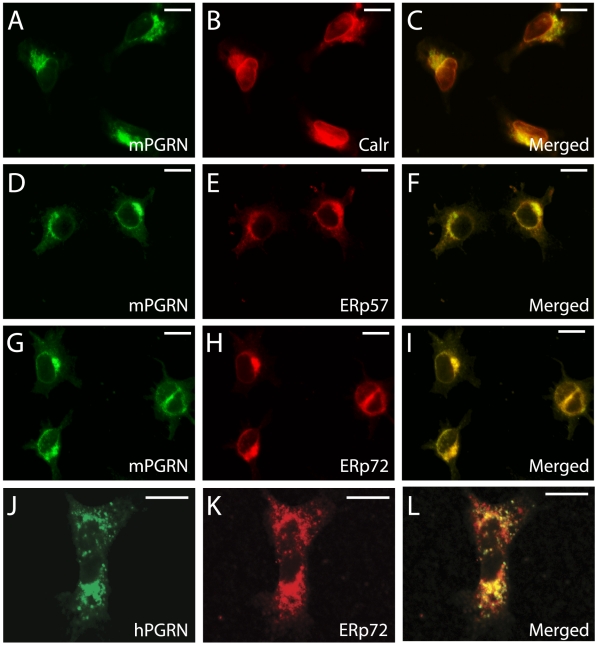
Co-localization of mPGRN-HA with its interacting proteins in HEK293 cells. (**A, D, G**) Subcellular localization of mPGRN in HEK293 cells. (**B**, Co-immunostaining of cells in (A) with anti-calreticulin (Calr) antibody. (**C**) the merged image of (A) and (B). (**E**) Co-immunostaining of cells in (D) with anti-ERp57 antibody. (**F**) The merged image of (D) and (E). (**H**) Co-immunostaining of cells in (G) with anti-ERp72 antibody. (**I**) Merged image of (G) and (H). The mPGRN antibody did not recognize endogenous hPGRN in HEK293 cells. (**J**) Subcellular localization of endogenous hPGRN in HEK293 cells. (**K**) Subcellular localization of endogenous ERp72 in HEK293 cells. (L) Merged image of (J) and (K) indicates that endogenous hPGRN also largely co-localizes with ERp72. Note that the size of scales bars in J–K are different from A–I and mPGRN is overexpressed in A–I and shows a stronger signal in the ER. Scale bars: A–I, 10 µm; J–L, 10 µm.

To confirm the biochemical findings, we examined the subcellular localization of PGRN by immunostaining. In HEK293 cells expressing mPGRN, an mPGRN-specific antibody showed that mPGRN co-localized with several ER proteins, such as calreticulin, ERp57, and ERp72 (Fig. 3, A–I). To confirm this finding, we also examined endogenous hPGRN expressed in HEK293 cells. Indeed, endogenous hPGRN is also co-localized with ERp72 (Fig. 3, J–L), raising the possibility that PGRN secretion may be a rate-limiting step.

### PGRN Is Expressed from Primary Cortical Neurons and Microglia But Not by Astrocytes

**Figure 4:**
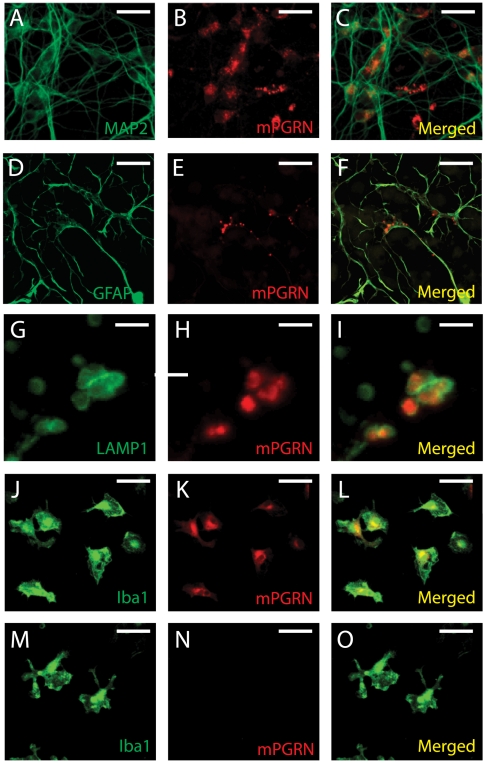
Expression of endogenous PGRN in different brain cell types. (**A–C**) mPGRN is expressed in MAP2-positive cultured mouse cortical neurons. (**D–F**) mPGRN is present in the processes of astrocytes in mixed brain cell cultures. (**G–I**) mPGRN seems to be localized in LAMP1-positive vesicles. (**J–L**) mPGRN is highly expressed in Iba1-positive cultured mouse microglia. (**M–O**) Anti-mPGRN specifically recognizes the endogenous mPGRN protein since the immunostaining signal is absence in Iba1-positive microglia (M) isolated from *GRN* knockout mice (N). Scale bar: 20 µm for all panels except G–I (3 µm).

To examine the expression patterns of PGRN in neural cells, we isolated primary cortical neurons and glial cells from E18 mouse embryos. Endogenous mPGRN was detectable in cultured MAP2-positive cortical neurons (Fig. 4, A–C), consistent with *in situ* analysis of mouse brains [18]. mPGRN was not detected in astrocytes in cultures without neurons but was present in granular structures in the processes of astrocytes in mixed cortical cell cultures (Fig. 4, D–F). Further immunostaining analysis indicated that these granular structures seemed to be inside vesicles expressing the lysosomal marker LAMP1 (Fig. 4, G–I), raising the possibility that astrocytes take up mPGRN secreted by neurons and transport it to lysosomal compartments.

**Figure 5:**
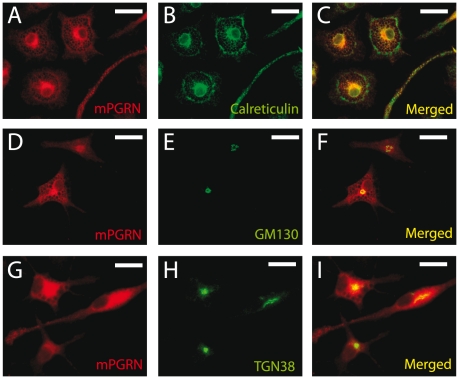
Subcellular localization of endogenous mPGRN in cultured primary microglia. (**A, D, G**) Immunostaining of endogenous mPGRN in cultured primary microglia. (**B**) immunostaining of the ER marker calreticulin in the microglia in (A). (**C**) Merged image of (A), and (B). (**E**) Immunostaining of the cis-Golgi marker GM130 in the microglia in (D). (**F**) Merged image of (D) and (E). (**H**) Immunostaining of the trans-Golgi marker TGN38 in the microglia in (G). (**I**) Merged image of (G) and (H). Scale bar: 20 µm.

mPGRN was prominently expressed in cultured microglia from postnatal day 3–4 mouse brains (Fig. 4, J–L). The staining signal was specific to mPGRN, since no signal was observed when the same antibody was used on microglia from *GRN* knockout mice [19] (Fig. 4, M–O). In microglia, mPGRN largely co-localized with the ER marker calreticulin (Fig. 5, A–C) and partly co-localized with the cis-Golgi marker GM130 (Fig. 5, D–F) and the trans-Golgi marker TGN38 (Fig. 5, G–I). Thus, a large fraction of PGRN remains in the intracellular ER-Golgi secretory pathway, suggesting that its posttranslational processing and secretion in neurons and microglia are inefficient and tightly regulated.

### ERp57 and ERp5 Promote mPGRN Secretion

**Figure 6:**
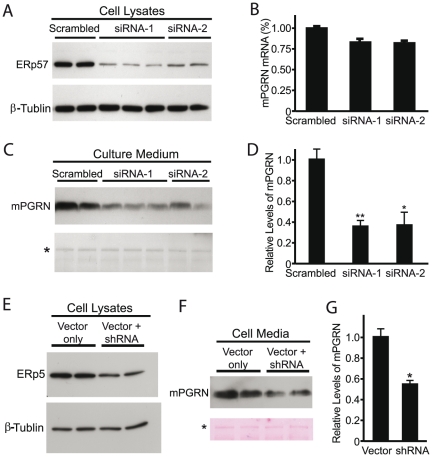
Loss of ERp57 activity caused reduced PGRN secretion. (**A**) Western blot analysis of the efficiencies of siRNA knockdown of ERp57 expression in stably transfected HEK293 cells expressing mPGRN. β-tubulin was used as the loading control. (**B**) qRT-PCR analysis of relative mPGRN mRNA levels after siRNA treatment. (**C**) mPGRN levels in the culture medium were measured by Western blot with anti-mPGRN antibody. *Asterisk* indicates a protein band of unknown identity visualized by staining of the Western blot membrane with Ponceau S to indicate equal loading. (**D**) Quantification of mPGRN levels in *C*. The values are mean ± SEM. *p<0.02, **p<0.002 vs. scrambled control. This experiment was repeated three times with similar results. (**E**) A vector-based shRNA construct reduced ERp5 expression level in stably transfected HEK293 cells expressing mPGRN. β-tubulin was used as the loading control. (**F**) Levels of secreted mPGRN were reduced in the culture medium of HEK293 cells with partial knockdown of ERp5. *Asterisk* indicates a protein band of unknown identity visualized by staining of the western blot membrane to indicate equal loading. (**G**) Quantification of the levels of mPGRN in *F*. Values are mean ± SEM. *p<0.02. This experiment was repeated three times with similar results.

PGRN expression is more than 60-fold higher in activated microglia than in resting microglia *in vivo* [17]. By using stably transfected HEK293 cells expressing mPGRN under control of the constitutively active CMV promoter, we were able to examine the specific effects of PGRN-interacting proteins on post-tanscriptional regulation of overexpressed mPGRN such as its secretion. Since BiP, GRP94, and calreticulin are all key ER chaperones essential for cell viability and mammalian development [20], we decided to examine identified PDI family proteins that exhibit distinct substrate specificities [21]–[23]. For instance, we used siRNA to knock down the expression of endogenous ERp57, a PDI protein that regulates the secretion of glycosylated proteins (Fig. 6A). Because PDI is an essential gene, we didn't perform similar analysis on this gene. Two independent siRNA sequences also effectively reduced ERp57 expression in HEK293 cells (Fig. 6A) with minimal effect on mPGRN mRNA expression, as shown by real time RT-PCR (Fig. 6B). Both siRNAs also reduced secretion of mPGRN in the culture medium (Fig. 6, C and D). This finding is consistent with reports that ERp57 is required for the proper secretion of several other substrates [22]. Examination of intracellular progranulin under native non-denaturing conditions showed one prominent mPGRN band that didn't reveal a difference in its folding state before and after ERp57 knockdown (data not shown), suggesting that misfolded PGRN due to blockage of its secretion is likely degraded. Indeed, immunostaining experiment didn't detect a difference in mPGRN subcellular localization after ERp57 knockdown (data not shown).

Another PGRN-interacting PDI member identified from our biochemical analysis is ERp5 (Table 1). Similar to ERp57, knockdown experiments show that ERp5 also promotes PGRN secretion (Fig. 6E–G), indicating their overlapping functions in this process.

## Discussion

PDI family proteins in the ER are responsible for the proper formation of intramolecular disulfide bonds through thiol-disulfide oxidation, reduction, and isomerization reactions on newly synthesized membrane proteins and secreted proteins [21]–[23]. Humans have 20 PDIs, which differ in the number and arrangement of thioredoxin-like domains, and the precise functions and substrate specificity for most of these PDIs are unclear. One of the most well-studied is ERp57, which is glycoprotein-specific and interacts with the lectin-like chaperones calnexin and calreticulin [23]. PGRN is a secreted and highly glycosylated protein with 88 cysteines [16], [24]. Thus, the identification of its physical interactions with PDI members and calreticulin in this study is not unexpected. Acute knockdown of ERp57 reduced PGRN secretion (Fig. 6), consistent with reports that ERp57 is essential for the proper folding of many membrane and secreted proteins and their transport through the ER-Golgi pathway [25]–[27]. Our findings here also indicate that progranulin is a previously unknown substrate for ERp5.

Our results also indicate that PGRN is regulated by a network of ER molecular chaperones as a novel substrate of ERp72. Although the exact molecular function of ERp72 is largely unknown, structural analyses suggest that its substrate specificity differs from that of PDI or ERp57 [28], [29]. ERp72 seems to have a narrow substrate specificity but exerts no detectable effects on the secretion of some tested substrates [27]. Indeed, we generated *ERp72* knockout mice and found that progranulin secretion from cultured neurons was not affected (unpublished data).

PGRN expression is markedly elevated in many physiological and pathological conditions, such as cell proliferation, wound healing, tumorigenesis, and brain injury [16], [17]. Of particular interest, the intracellular level of PGRN expression is more than 60-fold higher in activated microglia than in resting microglia in mouse brain [17]. This finding, together with other reports ([3], [4] and our analysis on subcellular localization of PGRN in culture, raises the possibility that PGRN folding and secretion are inefficient and tightly regulated. Thus, the molecular chaperones identified this study may help us understand the molecular machineries that regulate the posttranslational regulation of PGRN in certain pathological conditions.

In frontotemporal dementia patients with PGRN deficiency, one promising therapeutic approach is to increase production and/or secretion of PGRN from the remaining wildtype allele. Our findings raise the possibility that modulating the ER chaperone network to increase the level of extracellular PGRN may be a novel strategy for treating these patients.

## Materials and Methods

### Ethics Statement

All animal works have been conducted according to relevant national and international guidelines with the protocol (Docket # 2193–10) approved by the Institutional Animal Care and Use Committee (IACUC) of the University of Massachusetts Medical School.

### Animals and Cell Cultures

*PGRN^−/−^* mice were obtained from Kayasuga et al. [30]. Primary microglial cultures were obtained from *PGRN^−/−^* mice and their littermates at 3–4 days of age. Cerebral cortices were dissected and incubated in 0.25% trypsin for 20 min at 37°C. Cells were dissociated in plating medium (Eagle's MEM supplemented with 10% FBS, 0.45% glucose, 0.11 mg/ml sodium pyruvate, 0.2 mM glutamine, 1% penicillin/streptomycin) and plated in poly-L-lysine–coated flasks. Three hours later, the plating medium was replaced with DMEM containing 10% FBS and 1% penicillin/streptomycin. At day 10, microglia cells were harvested and plated on poly-L-lysine–coated coverslips and cultured for 2–3 days in macrophage serum-free medium (Gibco).

Primary cortical neurons were isolated from embryonic day (E) 18 mice as described for microglia cells, except that, cells were plated on poly-L-lysine–coated coverslips after dissociation and cultured for 7 days in Neurobasal medium supplemented with 2% B-27, 0.2 mM glutamine, and 1% penicillin/streptomycin.

### Immunocytochemistry

Cells were fixed in 4% paraformaldehyde (pH 7.4) for 10 min and incubated in 0.1% saponin and 3% BSA for 30 min. Next, cells were incubated in primary antibodies for 1 h at room temperature. The primary antibodies used were mouse anti-trans-Golgi network 38 (TGN38) (Affinity Reagents; 1∶100), mouse anti-GM130 (BD Bioscience; 1∶350), mouse anti-MAP2 (Sigma; 1∶200), mouse anti-LAMP1 (Stressgen; 1∶200), sheep anti-PGRN (R & D Systems, 1∶100), rabbit anti-α-Iba1 (Wako; 1∶350), rabbit anti-calreticulin (Sigma; 1∶250), rabbit anti-glial fibrillary acidic protein (Dako; 1∶1000), rabbit anti-ERp57 (1∶3000, a kind gift from Dr. D. Williams), goat anti-hPGRN (Alexis Biochemicals; 1∶100), rat anti-hemagglutinin (HA) (Roche; 1∶200). After three washes with PBS, the cells were incubated with AlexaFluor secondary antibodies (Invitrogen; 1∶200) for 1 h at room temperature. Immunostained cells were examined with a confocal microscope (D-Eclipse C1, Nikon).

### Chemical Cross-Linking and Immunoprecipitation

HEK293 cells (Invitrogen) expressing mPGRN-HA or vector were treated with Ca^2+^- and Mg^2+^-free PBS containing 1 mM disuccinyl suberate (DSS) (Pierce) or 1 mM bis[sulfosuccinimidyl] suberate (BS) (Pierce) for 30 min at room temperature. The cross-link reactions were quenched with 50 mM Tris (pH7.4), and cells were washed with PBS and lysed in immunoprecipitation buffer consisting of 50 mM Tris-HCl, 150 mM NaCl, 0.5% Triton X-100, 1 mM EDTA, 10 mM NaF, 15% glycerol, protease inhibitor cocktail (Pierce), and Halt phosphatase inhibitor cocktail (Pierce). Total protein lysate (1 mg) was incubated with 3 µg of anti-HA antibody (3F10, Roche) overnight at 4 °C and then with protein G-agarose for 3 h. Immunoprecipitates were washed with immunoprecipitation buffer and eluted in sample buffer. The samples were subjected to SDS-PAGE and immunoblot analysis or stained with silver stain for mass spectrometry analysis.

### Gene Knockdown and Western Blotting

mPGRN-HA stable cells were seeded on 12-well plates (2×10^5^ cells/well), transfected with negative control siRNA (Qiagen), siRNA directed against PDIA3/ERp57 (Qiagen) using Lipofectamine RNAiMax (Invitrogen) or vector-based PDIA6 siRNAs using Lipofectamine RNAiMax (Invitrogen). The medium was changed 48 h after transfection, and medium and cells were harvested 16 h later. The cells were lysed in RIPA buffer and centrifuged at 12,000 rpm for 10 min. Both lysates and medium were subjected to SDS-PAGE and immunoblot analysis with rabbit anti-ERp57 (1∶3000, a kind gift from Dr. D. Williams), anti-ERp5 (1∶2000, Abcam), sheep anti-mPGRN (R&D Systems; 1∶1000), mouse anti-β-actin (Sigma; 1∶5000), mouse anti-β-tubulin (Developmental Studies Hybridoma Bank; 1∶200), mouse anti-GAPDH (Millipore; 1∶2500), rat anti-HA (1∶2000, Roche). Immunoblots were developed with SuperSignal West Pico chemiluminescent substrates (Thermo Scientific).

### Molecular Cloning and RT-PCR

siRNA constructs were generated by cloning annealed oligonucleotides in pSUPER-GFP (Oligoengine) vector with *Bgl*II-*Xho*I enzyme sites. Total RNA was isolated with an RNeasy kit (Qiagen), and 500 ng of RNA were reverse transcribed to cDNA using TaqMan reverse transcription reagents (Applied Biosystems) following the manufacturer's instructions. Quantitative PCR was performed with SYBR Green Master Mix (Applied Biosystems) and 10 µM of forward and reverse primers. *GAPDH* was used as a control probe.
